# Disease Resurgence, Production Capability Issues and Safety Concerns in the Context of an Aging Population: Is There a Need for a New Yellow Fever Vaccine?

**DOI:** 10.3390/vaccines7040179

**Published:** 2019-11-08

**Authors:** Kay M. Tomashek, Mark Challberg, Seema U. Nayak, Helen F. Schiltz

**Affiliations:** Division of Microbiology and Infectious Diseases, National Institute of Allergy and Infectious Diseases, National Institutes of Health, 5601 Fishers Lane, Bethesda, MD 20892-9826, USA; MCHALLBERG@niaid.nih.gov (M.C.);

**Keywords:** yellow fever, vaccine, flavivirus, mosquito-borne, acute febrile illness

## Abstract

Yellow fever is a potentially fatal, mosquito-borne viral disease that appears to be experiencing a resurgence in endemic areas in Africa and South America and spreading to non-endemic areas despite an effective vaccine. This trend has increased the level of concern about the disease and the potential for importation to areas in Asia with ecological conditions that can sustain yellow fever virus transmission. In this article, we provide a broad overview of yellow fever burden of disease, natural history, treatment, vaccine, prevention and control initiatives, and vaccine and therapeutic agent development efforts.

## 1. Background 

Yellow fever (YF) is a potentially fatal disease caused by a member of the family Flaviviridae, genus *Flavivirus*, yellow fever virus (YFV) [1]. The disease is endemic in equatorial Africa, tropical areas of South America, eastern Panama and Trinidad. YFV is transmitted to humans and non-human primates (NHP) by *Aedes* spp. mosquitoes in Africa [2,3] and *Aedes* spp., *Haemagogus* spp. and *Sabethes* spp. mosquitoes in South America [4,5]. There are three transmission cycles for YFV: a sylvatic cycle involving NHP and *Aedes africanus, Haemagogus* spp. or *Sabethes* spp. mosquitoes in jungles with sporadic transmission to humans frequenting these areas; an intermediate cycle involving NHPs, humans and *Aedes* spp. mosquitoes in African savannah settings; and an urban cycle involving primarily *Aedes aegypti* mosquitoes and humans in cities. While the sylvatic and intermediate YFV transmission cycles account for most human disease, in the last decade there has been a resurgence in urban outbreaks [6]. Many of the recent African outbreaks, including in Uganda in 2010 [7,8], Ethiopia in 2013 [9], Angola in 2015 [10,11] and Nigeria in 2017 [12], were the first YF outbreaks in these countries in more than 10 years. Moreover, the Angola outbreak resulted in spread to the Democratic Republic of the Congo (DRC)[13], Mauritania and Kenya [14], as well as importation of disease into China by unvaccinated Chinese nationals who were infected while working in Angola [15,16]. In South America, a large urban outbreak was detected in 2016 in a non-endemic area of Brazil, and from 2016 to March of 2019 there were 2204 reported human cases or more cases than in the previous 20 years combined [17,18,19,20,21,22]. 

Collectively, these recent YF outbreaks have increased the level of concern about the disease and its potential to spread to non-endemic areas in Africa and Latin America and countries in Asia where YFV is absent [23,24]. Factors that may favor the spread of the disease and increase the likelihood of large urban outbreaks include more abundant vector populations over a wider geographic area [25], increasing urbanization, a highly mobile human population [21,26,27,28], an increase in the NHP population [29], and NHP displacement due to deforestation in the Amazon Basin and forested areas in equatorial Africa [24,30]. Low population immunity to YFV in neighboring endemic areas due to under vaccination may also be a contributing factor [31,32]. 

In response to outbreaks in Angola and DRC, in 2017, the World Health Organization (WHO), UNICEF, and Gavi, the Vaccine Alliance developed a new global initiative, the Eliminate Yellow Fever Epidemics (EYE) Strategy, in collaboration with 50 partners in support of 40 high-risk countries [33]. EYE focuses on preventing sporadic YF cases from developing into outbreaks, mitigating outbreaks, and preventing exportation once outbreaks are identified. The goal of EYE is to eliminate YF outbreaks globally by 2026 by vaccinating 1.4 billion people in 40 countries by supporting vaccination programs and campaigns and strengthening surveillance and laboratory capacity to detect, monitor and respond to YFV transmission. EYE built upon lessons learned from the WHO Yellow Fever Initiative which was created in collaboration with Gavi, the Vaccine Alliance and UNICEF in response to YF outbreaks in West Africa in the early 2000s. The Yellow Fever Initiative introduced YF vaccine into routine childhood immunization schedules in endemic countries, conducted mass preventive campaigns in risk areas, and established a global vaccine stockpile to respond to outbreaks. While initiative efforts successfully prevented YF outbreaks in West Africa, many of the recent African outbreaks have occurred in Central and East Africa.

## 2. Burden of Disease 

More than 900 million people reside in 35 African and 13 Central and South American countries where there is high to moderate risk of YFV transmission [33]. While the last estimate of YF incidence and mortality is from 1992 [34], 90% of YF cases reported to WHO via passive surveillance are still from Africa. One recent study estimated that there were 130,000 (95% CI 51,000–380,000) YF cases with fever and jaundice or hemorrhage in 2013, including 78,000 (95% CI 19,000–180,000) deaths in Africa [35]. In endemic areas of Africa, natural immunity accumulates with age, thereby putting infants and children at highest risk. In the Americas, the annual reported incidence of YF is typically less than 500 cases with the exception of 524 cases in 1995, 825 cases in 2017, and 1401 cases in 2018 [4,36]. Traditionally, most cases in the Americas were reported among unimmunized men thought to be exposed to YFV-infected mosquitoes while working in forested areas.

After being bitten by a YFV-infected mosquito, the incubation period is 2 to 9 days [37]. Most infected people develop subclinical infections or mild, self-limited clinically-apparent infections not requiring medical attention [38]. Classic YF has three stages. The first stage occurs while the patient is viremic and consists of nonspecific clinical features including fever, chills, generalized malaise, headache, red conjunctivae, photophobia, low back pain, myalgia, anorexia, nausea, vomiting, hepatomegaly, and epigastric and hepatic tenderness upon palpation. During this stage, patients are often leukopenic and have elevated serum transaminase levels, which may be predictive of ultimate disease severity [39,40,41]. After 3 to 4 days, patients enter a second 24 to 48-hour remission stage when they become afebrile and their symptoms lessen. Most patients remain afebrile and recover while about 12% (95% CI 5–26) develop severe disease and enter the third stage of illness [38]. During the third stage, the fever returns, and patients develop nausea, vomiting, epigastric pain, jaundice, oliguria, and hemorrhagic manifestations. Patients may develop metabolic acidosis and organ impairment involving the liver, pancreas, kidneys and cardiovascular system [42]. Neurologic complications may occur including seizures, encephalopathy, cerebral edema, and coma. The case fatality rate among patients with severe disease has been estimated to be 47% (95% CI 31–62)[38].

## 3. Immune Response after Natural Infection

Those who recover from YF likely have long-lasting immunity, however most data on immune response and antibody kinetics in YF are from vaccine models [43]. IgM antibodies are typically detected during the first week of illness, peak during the second week, and rapidly decline over the next sixty days. While IgM antibodies can persist after YF immunization, it is unknown if there is similar endurance after natural infection [44]. Neutralizing antibodies, which primarily consist of IgG antibodies, can remain for many years after immunization and natural infection, and can confer protection against re-infection [45,46,47,48]. There are no documented cases of repeated YFV infection, even in several historical cohorts [49]. Evidence also suggests that previous heterologous flavivirus exposure, for example from prior dengue virus infection, may provide partial cross protection against severe YF [43,50,51].

## 4. Treatment for Yellow Fever

The standard treatment for YF patients is supportive care, as there are currently no FDA-approved therapeutic agents to treat the disease or prevent disease progression despite decades of research and development investments [52]. Supportive care may include nutritional support, administration of intravenous fluids, and acetaminophen for pain and fever. Patients with severe disease require intensive care management to prevent and treat shock, metabolic acidosis, hypoglycemia, stress-induced gastritis, bleeding, hepatic dysfunction, renal failure, and secondary bacterial infections [53]. Patients should be protected from mosquito bites for five days after fever onset to avoid infecting mosquitoes.

Currently, the most promising therapeutic agents in early clinical development are nucleoside analogs that inhibit viral RNA-dependent RNA polymerases [54,55,56,57,58]. For example, galidesivir has in vitro and in vivo activity against a broad range of RNA viruses, including Ebola, Marburg and YFV [58,59]. A Phase 1 clinical trial to evaluate the safety, pharmacokinetics and anti-viral effects of galidesivir in patients with laboratory-confirmed YF is ongoing [60]. Numerous studies have identified other potential antiviral candidates; however, none have entered clinical trials. For example, sofosbuvir, a uridine analog used to treat hepatitis C, has shown antiviral activity against YFV in cell-based assays and rodent infection models [54,57]. Further preclinical development needs to be done for this and other candidates, and their clinical utility to treat YF remains untested.

A few monoclonal antibodies targeting the conformationally conserved envelop (E) protein Domain II or III involved in membrane fusion have shown promise with many demonstrating broad cross-activity against multiple flaviviruses including YFV [61,62,63,64,65,66]. One example of a promising, first-in-class monoclonal antibody for YF is TY014 developed by Tychan, Singapore [67]. TY014 was designed to neutralize multiple strains of YFV by binding to an epitope on the E protein on the surface of the virus, thereby preventing viral fusion to the host cell during viral entry [68]. TY014 will be evaluated in a Phase I clinical trial in healthy adult subjects to assess safety and the effect of TY014 on vaccine-associated viremia [67].

## 5. Disease Prevention

People living in or traveling to areas with risk for YFV transmission should avoid mosquito bites whenever possible [69]. Those who get YF should avoid mosquito bites for five days after fever onset to avoid infecting naive mosquitoes that can then transmit YFV to others. Persons ≥9 months old living in or traveling to areas with risk for YFV transmission should be vaccinated unless they have a contraindication to vaccination with a live-attenuated vaccine.

Preventing YF outbreaks is challenging for public health authorities for several reasons. First, public health officials have no control over the largely inaccessible sylvatic mosquito species that lay eggs in tree holes and the NHP reservoir [24]. Instead, epizootics among NHPs are monitored as sentinel events to enable early detection of human cases [70,71]. Second, *Aedes spp.* mosquitoes have become more abundant and geographically dispersed in recent decades, in part due to ineffective and unsustainable vector control strategies [72,73]. Despite limited evidence supporting the effectiveness of control strategies, novel mosquito modification methods combined with an updated integrated vector control program may be the most effective option to mitigate YFV transmission in urban areas [74,75]. Third, the disease is largely sub-clinical, so outbreaks may go undetected until severe cases are identified by surveillance. This allows the outbreak to become widespread before interventions, like mass vaccination campaigns, are initiated. Last, while the current YF 17D vaccine is thought to be effective [76], 80% of the population needs to be vaccinated in areas with risk for YFV transmission to prevent outbreaks. This level of coverage requires significant support and commitment to national vaccination programs and control strategies as well as the capacity to predict where YF outbreaks may occur and adapt strategies to expand coverage to emerging at-risk areas [77,78,79,80,81].

## 6. Yellow Fever Vaccines and Production Capacity

A live-attenuated YF vaccine was first developed in 1936 using an attenuated wild-type YFV strain that was isolated in 1927 from a Ghanaian patient named Mr. Asibi [82,83]. The Asibi 17D strain was passaged 53 times in monkeys, followed by serial passage in minced tissue preparations: mouse embryo (18 passages), whole chick embryo (58 passages), and whole chick embryo without brain and spinal cord (100 passages) [84]. During the passage series, the virus was assayed for neurotropism in mice by intracerebral injection, and pathogenicity in monkeys by intracerebral and extraneural administration. The 176th viral passage was selected to make vaccine sub-strains 17D-204 and 17DD because it was less lethal when delivered intracerebrally than a 114th passage strain, the first strain to lose pathogenicity in mice. A third sub-strain, which is a derivative of 17D-204, the 17D-213 sub-strain, was produced in 1977 and is a reference stock maintained by WHO for new manufacturers or emergency production [43,85].

Currently, six YF 17D vaccines are produced worldwide, all of which are derived from the Asibi strain. Four are WHO-prequalified YF vaccines which are used internationally for WHO/UNICEF vaccination campaigns: Bio-Manguinhos/FIOCRUZ (Brazil), YF 17-DD vaccine; Sanofi Pasteur (France), YF 17D-204 vaccine, Stamaril®; Institut Pasteur Dakar (Senegal), YF 17D-204 vaccine; and the Chumakov Federal Scientific Center (Russian Federation), YF 17D-204 vaccine. In addition, the Sanofi Pasteur (USA), YF 17D-204 vaccine, YF-Vax® is used in the U.S. and Canada, and China has a YF 17D-204 vaccine produced for domestic use by the China National Biotech Group. 

Even though there are several YF vaccine producers, two with large scale capability, supply has not been able to keep up with demand. Production capacity is limited because the vaccines are produced in embryonated eggs using a labor-intensive method that is relatively unchanged since 1945 [85]. As a result, only about 80 million doses are produced each year [86]. It is estimated that there are between 393.7 and 472.9 million people residing in YFV transmission risk areas who need to be vaccinated to achieve the recommended 80% population coverage threshold [33,87]. Moreover, the incidence of YF appears to be increasing not only in under-vaccinated areas but also in historically non-endemic areas [6]. When YF outbreaks occur, the vaccine supply is insufficient to meet the needs for mass vaccination campaigns [88]. For example, in an outbreak in Kinshasa in 2016, vaccine shortage led to the emergency use of a fractional (i.e., one-fifth) dose of YF 17-DD vaccine after the vaccine stockpile was depleted [89,90,91]. In 2018, a campaign using fractional doses of YF vaccine was initiated in Brazil in response to urban outbreaks in São Paulo and Rio de Janeiro. While fractional dosing may be employed during public health emergencies, broader application is not recommended because of insufficient data regarding the immunogenicity of fractional doses in infants, young children and human immunodeficiency virus (HIV)-infected persons living in endemic areas [32,92,93].

## 7. Safety Profile of Yellow Fever Vaccines 

Serious adverse events (SAE) including deaths occur following administration of YF vaccine [27,94,95,96], and the frequency of vaccine-related SAEs for YF vaccine is comparable to the rate of vaccine-associated paralytic poliomyelitis with oral polio vaccine [97,98]. In addition, because YF vaccine is a live-attenuated vaccine produced in eggs, there are several contraindications and precautions to its administration [99]. Experts have called for the development of a safer YF vaccine ever since viscerotropic disease, an acute illness resembling severe wild-type disease but with higher lethality, was identified [96,97]. However, until we have a safer vaccine, healthcare providers and public health officials will have to determine if the benefit outweighs the risk among travelers to endemic areas and for populations living in non-endemic areas bordering endemic areas. 

A review of U.S. Vaccine Adverse Event Reporting System (VAERS) data from 2007–2013 found 3.8 SAEs per 100,000 vaccine doses [94], while a review of passive surveillance data from several non-endemic countries found rates ranging from 1.3 to 5.1 SAE per 100,000 vaccine doses [98]. The rate is known to increase with age such that 60–69-year old vaccine recipients had a rate of 6.5 SAEs per 100,000 doses while those over 70 years old had a rate of 10.3 [94]. The three primary SAEs include anaphylaxis (0.2–1.8 per 100,000 doses), neurologic disease (0.1–3.9 per 100,000 doses) and viscerotropic disease (0.07–0.4 per 100,000 doses) due to 17D virus infection of the liver and visceral organs [98,100].

Yellow fever vaccine-associated viscerotropic disease (YEL-AVD) was first described in 2001, although cases as far back as the 1970s have been identified retrospectively [43]. While YEL-AVDs are rare events, the case fatality rate is >60%. YEL-AVD typically presents about 4 days after the first YF vaccine dose is administered and has a natural history similar to severe wild-type disease. YEL-AVD occurrence is thought to be due to underlying host susceptibility rather than the vaccine virus reverting to a virulent phenotype [101]. Risk factors for YEL-AVD include thymus disorders, thymectomy, autoimmune diseases (e.g., systemic lupus erythematosus), host genetics, and advanced age; vaccine recipients ≥60 years have an incidence of 1.2 per 100,000 doses [100,102,103,104]. Older individuals may be at increased risk due to immune senescence and/or underlying chronic diseases. In fact, one study found that older first-time 17D vaccine recipients had a delayed antibody response and a longer duration of viremia than younger vaccine recipients [105]. Findings from animal models of YF disease support the hypothesis that an impaired innate immune system may allow dissemination of the 17D strain [106,107].

YFV is a neurotropic virus and while YF vaccine is attenuated, it is a live vaccine that replicates in the host and produces a brief viremia that has potential for neuroinvasion in a susceptible host. In addition, a recent study demonstrated that Schwann cells can support sustained replication of the 17D strain [108]. Encephalitis and meningoencephalitis associated with 17D vaccine administration in infants was first recognized in 1952 and led to the contraindication against vaccination of infants <6 months old [109]. Since then, vaccine-associated neurotropic disease (YEL-AND) has come to include not only neurologic disease due to direct viral invasion of the central nervous system (e.g., encephalitis and meningitis) but also autoimmune-mediated demyelination and neuropathy including Guillain-Barre´ syndrome, acute disseminated encephalomyelitis, myelitis, and cranial neuropathies. YEL-AND typically occurs 2-56 days after the first YF vaccine dose is administered, and the disease is generally not fatal. YEL-AND tends to affect young infants, immunocompromised individuals, and the elderly. The incidence of YEL-AND is 2.2 cases per 100,000 vaccine doses among vaccine recipients ≥60 years old [110,111,112]. In 2009, the first case of encephalitis in an infant was confirmed to be caused by YF vaccine virus transmitted via breastmilk [113,114,115]. As a result, breastfeeding women should not receive YF vaccine except when exposure to YFV cannot be avoided or travel cannot be postponed to YF endemic or epidemic areas.

## 8. Yellow Fever Vaccine Effectiveness

While there were no randomized, placebo-controlled clinical trials done to evaluate the efficacy or effectiveness of YF 17D vaccine prior to its use in humans, pre-clinical studies found that vaccination protected NHPs against a lethal challenge of YFV [43]. There is no evidence that vaccine effectiveness has played a role in the recent outbreaks as there is still only a single YFV serotype and while the lineages between South America and Africa continue to diverge, they are still very closely related [116]. Moreover, the majority (>90%) of vaccinated people develop neutralizing antibodies by 28 days after vaccination, and vaccine failures are thought to be rare [117]. A recent paper described 23 vaccine failures identified after >540 million doses of YF vaccine were administered, and five of these cases occurred before protective antibodies titers would be expected to develop after vaccination [76]. Observations and reports from the field support its effectiveness including the prevention of laboratory-acquired YFV infections among vaccinated staff and the decline in YF incidence after vaccination campaigns with subsequent cases identified only among unvaccinated individuals [43].

Not only is it considered an effective vaccine, but data suggests that a single dose of YF vaccine may confer life-long protection against YF disease [117,118]. In 2013, the WHO Strategic Advisory Group of Experts (SAGE) working group for YF vaccination recommended that the 2003 WHO position on 17D booster schedules and international health regulations be revised to remove the 10-year booster dose requirement [118]. In 2015, the Advisory Committee on Immunization Practices (ACIP) issued similar guidance on the adequacy of a single dose of YF vaccine for most travelers. However, ACIP also recommended that at-risk laboratory personnel and certain travelers receive additional doses of YF vaccine [76]. Additionally, the impact of no booster vaccination is being investigated among children as there is little evidence to support long-term persistence of protective immunity in primary vaccinated children [119].

## 9. Yellow Fever Vaccine Candidates

New YF vaccines that are produced using modern technologies are needed to address the limited production capacity of existing vaccines especially considering the expanding geographic distribution, threat of re-urbanization and possibility of introduction into YFV-naïve Asia [120]. In addition, new YF vaccines would ideally have a better safety profile for infants, breastfeeding women, immunocompromised individuals, and older people [98]. While vaccines are being developed, dose-sparing methods are being investigated [121,122]. One method, intradermal administration of YF vaccine, mimics the natural route of transmission and takes advantage of the skin dendritic cells role in the immune response [123,124]. In one study, the immunogenicity of a fractional (one-fifth) intradermal dose of YF 17D vaccine was not inferior to that of a full subcutaneous dose.

One approach being taken to address these issues is to produce an inactivated whole virus vaccine using the 17D virus produced in cell culture [120,125,126,127,128,129]. Such a vaccine would provide scalable production and would likely not have the adverse effects associated with viral replication. A candidate vaccine comprised of 17D virus inactivated by treatment with beta-propiolactone (BPL) was shown to protect mice against a lethal YFV challenge [129]. In a Phase 1 clinical trial, two doses of this vaccine elicited neutralizing antibody titers that were like those obtained with live-attenuated 17D vaccine, although the durability of this immune response is not yet known [130]. Another similar inactivated whole virus vaccine that is under development uses a new hydrogen peroxide-based inactivation method that preserves neutralizing epitopes on the virion better than conventional methods (Mark Slifka, personal communication) [131]. It is possible that this improved inactivation method will result in a vaccine that induces protective immunity after a single dose, but that remains to be determined. 

A second approach to produce a safer YF vaccine is to express the YFV pre-M and E proteins either as recombinant proteins or in another safer viral vector. The most advanced vaccine of this type utilizes the replication-defective Modified Vaccinia Ankara (MVA) vector [132]. This vaccine has been shown to induce protective immunity in small animal models and is currently being evaluated in a Phase I trial in humans [133].

## 10. Summary Points

YF is a potentially fatal, mosquito-borne viral disease that appears to be experiencing a resurgence in endemic areas in Africa and South America and spreading to non-endemic areas despite an effective vaccine. With increasing globalization and mobility, YFV has the potential to spread to YFV-naïve regions in Asia that have ideal ecological conditions to sustain viral transmission.

In response to the recent resurgence in YF, the EYE strategy was developed by WHO, Gavi, the Vaccine Alliance and UNICEF in collaboration with 50 other partners to vaccinate at-risk populations, mitigate outbreaks and prevent the spread of the disease. In order to do this, 80% of the population in risk areas need to be vaccinated, surveillance and laboratory capacity strengthened, and newer methods to predict emerging risk areas developed and utilized. This means that an adequate supply of YF vaccine needs to be made available.

YF 17D vaccines are effective and safe for most people. However, with an aging population throughout much of the tropics, a new vaccine with a better safety profile in person >60 years is desirable. In addition, a new vaccine with robust production capacity is needed to keep up with the demand as demonstrated by the recent outbreaks in DRC and Brazil in which fractional dosing had to be used. Two vaccine development approaches are being investigated to address production capacity and safety: inactivated whole virus vaccines using the 17D virus produced in cell culture and replication-defective MVA vector vaccine expressing the YFV pre-M and E proteins.
